# Germline Polymorphisms in the Nuclear Receptors PXR and VDR as Novel Prognostic Markers in Metastatic Colorectal Cancer Patients Treated With FOLFIRI

**DOI:** 10.3389/fonc.2019.01312

**Published:** 2019-11-26

**Authors:** Elena De Mattia, Jerry Polesel, Rossana Roncato, Adrien Labriet, Alessia Bignucolo, Eva Dreussi, Loredana Romanato, Michela Guardascione, Angela Buonadonna, Mario D'Andrea, Eric Lévesque, Derek Jonker, Félix Couture, Chantal Guillemette, Erika Cecchin, Giuseppe Toffoli

**Affiliations:** ^1^Clinical and Experimental Pharmacology, Centro di Riferimento Oncologico di Aviano (CRO) IRCCS, Aviano, Italy; ^2^Unit of Cancer Epidemiology, Centro di Riferimento Oncologico di Aviano (CRO) IRCCS, Aviano, Italy; ^3^Pharmacogenomics Laboratory, Centre Hospitalier Universitaire de Québec (CHU de Québec) Research Center and Faculty of Pharmacy, Laval University, Quebec City, QC, Canada; ^4^Medical Oncology Unit, Centro di Riferimento Oncologico di Aviano (CRO) IRCCS, Aviano, Italy; ^5^Medical Oncology Unit, “San Filippo Neri Hospital”, Rome, Italy; ^6^CHU de Québec Research Center and Faculty of Medicine, Laval University, Quebec City, QC, Canada; ^7^Division of Medical Oncology, Department of Medicine, Ottawa Hospital, University of Ottawa, Ottawa, ON, Canada

**Keywords:** pharmacogenetics, PXR, VDR, survival, FOLFIRI, colorectal cancer

## Abstract

Nuclear receptors act as mediators of cancer-related inflammation and gene expression. They have a regulatory effect on genes encoding proteins related to drug adsorption, distribution, metabolism, and excretion. The aim of the present study was to highlight novel prognostic markers among polymorphisms in genes encoding for nuclear receptor proteins and inflammation-related cytokines in patients treated with a FOLFIRI regimen. This study included two independent cohorts comprising a total of 337 mCRC patients homogeneously treated with first-line FOLFIRI. Genotyping of 246 haplotype-tagging polymorphisms in 22 genes was performed using bead array technology. The *NR1I2* (PXR)-rs1054190 and *VDR*-rs7299460 polymorphisms were significantly associated with patient overall survival (OS). A detrimental effect of the *NR1I2* rs1054190-TT genotype on OS was observed in both the discovery and replication cohorts (HR = 6.84, *P* = 0.0021, *q*-value = 0.1278 and HR = 3.56, *P* = 0.0414, respectively). Patients harboring the *NR1I2* rs1054190-TT genotype had a median OS of 9 months vs. 21 months in patients with C-allele (*P* < 0.0001 log-rank test). *VDR* rs7299460-T was consistently associated with a longer OS in both cohorts (discovery: HR = 0.61, *P* = 0.0075, *q*-value = 0.1535; replication: HR = 0.57, *P* = 0.0477). Patients with the *VDR* rs7299460-T allele had a median OS of 23 months compared to 18 months in those with the CC genotype (*P* = 0.0489, log-rank test). The *NR1I2*-rs1054190 polymorphism also had an effect on the duration of progression-free survival, consistent with the effect observed on OS. Two novel prognostic markers for mCRC treated with FOLFIRI were described and, if validated by prospective trials, have a potential application in the management of these patients.

## Introduction

FOLFIRI (irinotecan, bolus and continuous-infusion fluorouracil, leucovorin) represents one of the key regimens for the treatment of metastatic colorectal cancer (mCRC) due to the significant survival advantage reported by clinical trials as both pre-treated mCRC and first-line therapy (1, 2). Furthermore, the more recent combination of FOLFIRI with molecularly targeted anticancer agents, such as the anti-angiogenic bevacizumab or aflibercept (3, 4) and anti-EGFR agents cetuximab or panitumumab (5, 6), has further improved the efficacy of mCRC therapy.

However, despite the great advantage in patient survival obtained with the new treatment regimens, a remarkable inter-individual heterogeneity in therapy outcome still constitutes a crucial problem in mCRC management. Moreover, with the increasing number of therapeutic options, the selection of the most appropriate first-line treatment for mCRC becomes a complex issue influencing the course of therapy, and likely patient survival (7). Therefore, the identification of genetic markers that predict which patients will benefit from a specific intervention could significantly impact decision-making and therapeutic planning.

In the last few years, pharmacogenetics has been largely applied to the personalization of CRC treatment, specifically focusing on the genetic variability in adsorption, distribution, metabolism, and excretion (ADME) genes (8, 9). These research efforts have led to the validation of UDP glucuronosyl transferase family 1 member A1 (UGT1A1)^*^28 and some *dihydropyrimidine dehydrogenase (DPYD)* variants as predictive markers of irinotecan- or fluoropyrimidine-related toxicity and to its translation into specific clinical guidelines (10). However, the impact of germline ADME-related polymorphisms on the anti-tumor efficacy of the treatment is still questionable (8, 11).

Inflammation is a condition strictly linked to CRC development and progression, and it was recently reported to play a crucial role in ADME gene expression, including cellular transporters and phase I/II enzymes. This gene expression control is mediated by some transcriptional factors, including the nuclear receptors (NRs), whose activity is controlled by pro-inflammatory cytokine-induced signaling pathways, with a demonstrated impact on drug bioavailability and efficacy (12–15). These results have opened up a novel field of investigation that focuses on the contribution of inherited genetic variability in transcriptional regulators and inflammation cascade genes to the inter-individual differences in pharmacological profiles and therapeutic outcomes. In this context, significant associations between some genetic variants in *signal transducer and activator of transcription 3* (*STAT-3), vitamin D receptor (VDR)*, and *hepatocyte nuclear factor 1 alpha (HNF1A)* with the clinical outcomes of FOLFIRI were previously reported by our group (16, 17).

In the present study, we adopted a tagging polymorphism (TagSNP) approach to evaluate the overall variability of 22 transcriptional regulators and pro-inflammatory cytokines impacting FOLFIRI-related ADME genes to address the effect of these markers on overall survival (OS) in mCRC patients receiving the FOLFIRI regimen. The genetic variants that emerged as predictors of OS were further evaluated in relation to progression-free survival (PFS). The aim of this study, adopting a discovery/replication design, was to define potential novel genetic markers of survival in mCRC patients treated with FOLFIRI that could be considered to guide treatment decisions.

## Patients and Methods

### Patient Cohorts and Treatment

The study includes a total of 337 mCRC patients undergoing first-line FOLFIRI treatment and sub-grouped into discovery and replication cohorts. The previously described (18, 19) discovery cohort included prospectively enrolled North-Eastern Italian patients homogenously treated between February 2002 and November 2005 (18). OS data were available for all 250 eligible patients included in the study, whereas information on PFS was missing for 21 patients. Patients were treated with either a Tournigand-modified FOLFIRI regimen (20) (>90% of total) or FOLFIRI regimen based on a 180 mg/m^2^ intravenous dose of irinotecan. Details on eligibility criteria and treatment modalities, as well as the procedures for evaluating efficacy and data collection were published previously (18). Criteria for therapy delay/discontinuation were reported previously (18). The replication cohort included 90 patients recruited from 2003 to 2012 at three medical centers in eastern Canada (21). All patients received a 180 mg/m^2^ intravenous dose of irinotecan in FOLFIRI regimen every 2 weeks. Details on eligibility, treatment modalities, and clinical data were documented elsewhere (17, 21). In both cohorts, survival data were obtained through an active follow-up.

An additional cohort of 74 Eastern Canadian mCRC patients was considered to perform an exploratory analysis of the effect of the discovered genetic markers on the clinical outcome of patients treated with FOLFIRI plus bevacizumab. More details were previously reported (17, 21).

All patients in the study were self-reported Caucasian. The study protocol complied with the ethical guidelines of the 1975 Declaration of Helsinki. The protocol was approved by the Comitato Etico Indipendente-Centro di Riferimento Oncologico di Aviano and the CHU de Quebec ethics committees. All patients provided written informed consent for genetic analysis before entering the study. All experiments were carried out in accordance with the relevant guidelines and regulations of Centro di Riferimento Oncologico di Aviano and the CHU de Québec.

### Marker Selection

The candidate gene and polymorphism selection was already described in detail elsewhere (16). Briefly, transcriptional controllers and cytokines clearly implicated in the regulation of drug-related transporters and metabolic enzymes during inflammation were selected by a literature search (PubMed-MEDLINE). Genetic variants for each candidate gene were chosen successively using the TagSNP approach, covering the genetic diversity of the targeted genes. A set of 246 molecular markers in 22 candidate genes encoding NRs (*PXR, LXR-A/B, FXR, RXR-A/B/G, CAR, VDR, PPAR-A/G/D, HNF4A, HNF1A*), transcription factors and related pathways (*STAT-3, NF-kB1, IKBKB, CHUK*), and key pro-inflammatory cytokines (*TNF, IL-1B, IL-6, IFN*γ) were selected and introduced into the pharmacogenetic analysis (16).

### Genetic Analysis

#### Discovery Set

Genomic DNA was extracted from peripheral blood using the High Pure PCR Template Preparation Kit (Roche Diagnostics GmbH, Mannheim, Germany). DNA samples were genotyped using the Illumina BeadXpress platform based on Golden Gate chemistry. A 192-plex and 48-plex Illumina VeraCode GoldenGate Genotyping Assay (Illumina, Inc., San Diego, CA) was developed using the Assay Design Tool (ADT) available through Technical Support on the Illumina website (https://illumina.com). Details about the workflow for assay design, laboratory sample processing, data analysis, and quality control were reported previously (16). The genetic polymorphisms that did not pass quality control in the BeadXpress workflow were tested using an allelic discrimination method by predesigned TaqMan SNP genotyping assays. All commercial TaqMan assays were purchased from Applied Biosystems (https://www.appliedbiosystems.com) and the analyses performed using the Applera TaqMan Universal Master mix on an ABI 7500 (AB Applied Biosystems, Foster City, CA) according to the manufacturer's instructions. Positive and negative control samples were included in each analysis.

#### Replication Set

Polymorphisms to be tested in the replication cohort were genotyped by the Canadian research team using iPLEX matrix-assisted laser desorption/ionization time-of-flight mass spectrometry (Sequenom, San Diego, CA, USA). Negative controls and a 5% random sample duplicate population were used to ensure the robustness and reproducibility of the assay. All extension primers and PCR assays were designed using Spectro DESIGNER software (Sequenom, San Diego, CA, USA). Markers that could not be sequenced due to poor primer design or because they were located in duplicated regions were replaced with TagSNPs in complete linkage disequilibrium (LD; *r*^2^ = 1.00).

#### FOLFIRI Plus Bevacizumab Set

The polymorphisms identified as prognostic markers in this study and that need to be tested in the FOLFIRI plus bevacizumab cohort were genotyped using the iPLEX matrix-assisted laser desorption/ionization time-of-flight mass spectrometry (Sequenom, San Diego, CA, USA) as describe above.

### Statistical Analysis

The study design can be summarized in three main steps. The first step consisted of the selection of potential markers of OS (*P* < 0.01; *q*-value < 0.2) in the discovery cohort. Considering that significant polymorphisms in the discovery set were validated in an independent case series in the subsequent step, the *q*-value (22) was set to 0.20 to avoid the loss of potential relevant markers. In the second step, these selected polymorphisms were tested using the same genetic model in the independent replication cohort in order to find concordant [i.e., similar hazard ratio (HR)] and significant (i.e., *P* < 0.05) associations. The third step consisted of investigating a possible association between the replicated markers and PFS. An exploratory analysis on a cohort of patients treated with FOLFIRI plus bevacizumab was also performed to test the impact of the selected genetic markers on survival and risk of diseases progression.

The OS probabilities according to genetic polymorphisms were estimated by the Kaplan-Meier method and survival differences tested using the log-rank test (23). Time at risk was calculated from treatment initiation to death, progression (for PFS only), or last follow-up, whichever came first. The association between candidate polymorphisms and OS/PFS was evaluated by calculating the HR of death and corresponding 95% confidence interval (CI) in a Cox proportional hazards model (24). HRs were adjusted for gender, age, cancer site, stage at diagnosis, radical surgery, and adjuvant chemotherapy. Dominant, recessive, and additive genetic models were considered for each polymorphism by combining heterozygous with homozygous genotypes; the best-fitting genetic model was selected according to the Wald chi-squared test.

To determine the putative effect of the polymorphisms, a functional prediction was performed using the online software ENCODE-HaploReg v4.1 (https://pubs.broadinstitute.org/mammals/haploreg/haploreg.php), and RegulomeDB v1.1 (http://www.regulomedb.org/).

## Results

### Patients and Genotyping

The analytical revision and quality control of the genetic data obtained by genotyping were described in detail elsewhere (16). Three of the 250 samples in the discovery set were excluded from the study because they did not reach the fixed call rate threshold of 90%. Genotype data were available for 247 patients constituting the final discovery set. All 90 samples constituting the replication set were genotyped successfully. The main demographic and clinical characteristics of the two study populations (discovery and replication cohorts) are reported in Table 1.

**Table 1:** Socio-demographic and clinical characteristics of patients enrolled in the discovery and replication cohort.

	**Discovery cohort (*****n*** **=** **247)**		**Replication cohort (*****n*** **=** **90)**	
	***N***	**(%)**	***N***	**(%)**
**Gender**				
Male	160	(64.8)	60	(66.7)
Female	87	(35.2)	30	(33.3)
**Age (Years)**				
<55	62	(25.1)	23	(25.6)
55–59	32	(13.0)	16	(17.8)
60–64	52	(21.1)	17	(18.9)
≥65	101	(40.9)	34	(37.8)
**Cancer site**				
Right colon	78	(31.6)	22	(24.4)
Left colon/Rectum	169	(68.4)	61	(67.8)
Colon, NOS	0	(0.0)	8	(7.8)
**Stage at cancer diagnosis**				
I-II	25	(10.1)	–	
III	65	(26.3)	–	
IV	157	(63.6)	–	
**Radical surgery**				
No	50	(20.2)	–	
Yes	197	(79.8)	–	
**Adjuvant therapy**				
No	165	(66.8)	–	
Yes	82	(33.2)	–	
Median follow-up (Q1-Q3)	15 (10–24)		19 (12–32)	
**Overall survival (95% CI)**				
1 year	77.0% (71.0–82.0%)		76.7% (61.3–80.3%)	
2 years	44.7% (37.2–51.9%)		41.9% (31.4–52.0%)	
3 years	24.5% (16.1–33.9%)		20.9% (13.1–30.0%)	

*NOS, Not otherwise specified*.

### Markers of Overall Survival

All available 247 (discovery set) and 90 (replication set) patients were evaluable for the association between polymorphisms and OS. At the end of the study follow-up, 119 patients in the discovery cohort (48%) were still alive, and 128 patients (52%) were dead. In the replication cohort, 18 patients (20%) were still alive, and 72 patients (80%) were dead at follow-up. The survival information for the two study populations (discovery and replication cohorts) is reported in Table 1.

Each polymorphism was tested by Cox regression analysis for an association with OS. The results are summarized in Table 2. In the discovery cohort, 11 polymorphisms in genes encoding seven NRs (HNF4A, PXR, CAR, PPARA, PPARD, PPARG, VDR) and two transcription factors (STAT-3, NFkB1) were significantly (*P* < 0.01 and *q* <0.20) associated with OS. Of the 11 markers significantly associated with OS, 8 were associated with an increased risk of death, with HRs ranging from 1.63 to 37.6, and the remaining 3 were associated with a lower risk of death, with HRs ranging from 0.55 to 0.61.

**Table 2:** Hazard ratios (HR) and 95% confidence interval (CI) for death in the discovery (*n* = 247 mCRC patients) and replication (*n* = 90 mCRC patients) cohorts according to gene polymorphisms (SNP).

**Genes**	**SNP**	**Base change**		**Discovery set**			**Replication set**	
			**Model**	**HR (95% CI)^a^**	***P*-value**	***q*-value^c^**	**HR (95% CI)^b^**	***P*-value**
*HNF4A*	rs3212208	T > C	Rec	20.3 (2.57–161.0)	0.0043	0.1368	0.70 (0.09–5.23)	0.7256
*NFKB1*	rs3774934	G > A	Dom	0.55 (0.36–0.86)	0.0079	0.1535	1.04 (0.47–2.31)	0.9176
***NR1I2*** **(PXR)**	**rs1054190**	**C** **>** **T**	**Rec**	**6.84 (2.00**–**23.4)**	**0.0021**	**0.1278**	**3.56 (1.05**–**12.1)**	**0.0414**
*NR1I2* (PXR)	rs6784598	C > G	Dom	1.70 (1.16–2.50)	0.0067	0.1535	1.38 (0.80–2.38)	0.2537
*NR1I3* (CAR)	rs4073054	T > G	Rec	2.00 (1.29–3.09)	0.0020	0.1278	0.82 (0.38–1.80)	0.6241
*PPARA*	rs4253655	G > A	Add	1.63 (1.15–2.31)	0.0066	0.1533	0.76 (0.45–1.26)	0.2830
*PPARD*	rs4713854	A > C	Rec	27.0 (3.0–242.7)	0.0032	0.1278	1.36 (0.18–10.2)	0.7683
*PPARG*	rs7626560^§^	C > T	Rec	4.71 (1.81–12.3)	0.0015	0.1278	1.51 (0.58–3.90)	0.3954
*STAT3*	rs17593222	C > G	Rec	37.6 (4.41–320.4)	0.0009	0.1278	0.88 (0.11–7.09)	0.9045
*VDR*	rs4760648	C > T	Dom	0.57 (0.39–0.83)	0.0035	0.1278	0.64 (0.39–1.06)	0.0837
***VDR***	**rs7299460**	**C** **>** **T**	**Dom**	**0.61 (0.43**–**0.88)**	**0.0075**	**0.1535**	**0.57 (0.33**–**0.99)**	**0.0477**

*Only the associations with P-value < 0.01 and q-value < 0.20 are reported for the discovery set. Replicated markers are in bold*.

a *Estimated from Cox model, adjusted for gender, age, cancer site, stage at diagnosis, radical surgery, and adjuvant chemotherapy*.

b *Estimated from Cox model, adjusted for gender, age, and cancer site*.

c *FDR-adjusted P-value*.

§ *rs7626560 was replace in the replication set by rs13099078 which is in complete Linkage Disequilibrium (r^2^ = 1)*.

Two of the 11 markers, *NR1I2*-rs1054190 and *VDR*-rs7299460, were successfully replicated (*P* < 0.05) in the Canadian cohort applying the same genetic model. According to a recessive model, the T allele at *NR1I2*-rs1054190 was significantly associated with worse OS in the discovery (HR = 6.84, *P* = 0.0021, *q*-value = 0.1278) and replication (HR = 3.56, *P* = 0.0414) cohorts. In contrast, according to a dominant model, the T allele at *VDR*-rs7299460 was associated with longer OS in the discovery (HR = 0.61, *P* = 0.0075, *q*-value = 0.1535) and replication (HR = 0.57, *P* = 0.0477) cohorts.

The genotype distribution of these markers in the discovery and replication cohorts is reported in Supplementary Table S1. The minor allele frequencies (MAFs) were consistent with the data reported for the Caucasian population (http://www.ncbi.nlm.nih.gov/snp). Deviation from Hardy–Weinberg equilibrium was tested for each polymorphism in both the discovery and replication cohorts by χ^2^ test, and no deviation was found (*P* < 0.05) except for the variant *PPARA* rs4253655 in the discovery cohort.

**Figure 1:**
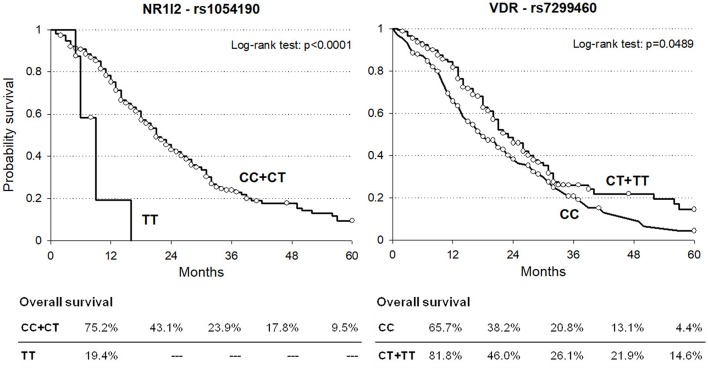
Kaplan-Meier estimates of overall survival according to selected *NR1I2*-rs1054190 and *VDR*-rs7299460 polymorphisms in combined discovery and replication cohorts (*n* = 337).

Kaplan-Meier curves for OS according to *NR1I2*-rs1054190 or *VDR*-rs7299460 polymorphisms in the combined discovery and replication cohorts are shown in Figure 1. Regarding *NR1I2*-rs1054190, patients carrying the minor allele homozygous rs1054190-TT genotype had a median OS of 9 months, compared to CC or CT carriers, who had a median OS of 21 months (*P* < 0.0001 log-rank test). In particular, among the eight patients harboring the rs1054190-TT genotype, only one was still alive 1 year from drug initiation, and no patient was still alive after 2 years (two patients were lost at follow-up, Supplementary Table S2). Regarding the *VDR*-rs7299460 polymorphism, patients with the minor allele homozygous rs7299460-TT or heterozygous rs7299460-TC genotypes had a median OS of 23 months, compared to those with the CC genotype, who had a median OS of 18 months (*P* = 0.0489, log-rank test).

### Markers of Time to Progression

Next, the association between *NR1I2*-rs1054190 and *VDR*-rs7299460 polymorphisms and PFS was tested (Table 3). At the end of the study follow-up, 203 out of 229 assessable patients (89%) in the discovery cohort had tumor progression or were dead (18). In the replication cohort, 82 out of 90 patients (91%) had tumor progression or were dead.

**Table 3:** Hazard ratios (HR) and 95% confidence interval (CI) for progression in the discovery (*n* = 229 mCRC patients) and replication (*n* = 90 mCRC patients) cohorts according to gene polymorphisms (SNP).

**Genes**	**SNP**	**Base change**	**Discovery set**			**Replication set**	
			**Model**	**HR (95% CI)^a^**	***P*-value**	**HR (95% CI)^b^**	***P*-value**
*NR1I2* (PXR)	rs1054190	C > T	Rec	2.65 (1.04–6.75)	**0.0413**	2.67 (0.80–8.89)	0.1087
*VDR*	rs7299460	C > T	Dom	0.93 (0.70–1.23)	0.5952	0.64 (0.38–1.06)	0.0843

*Only the markers significantly associated with overall survival were considered. Associations with P-value < 0.05 are evidenced in bold*.

a *Estimated from Cox model, adjusted for gender, age, cancer site, stage at diagnosis, radical surgery, and adjuvant chemotherapy*.

b *Estimated from Cox model, adjusted for gender, age, cancer site*.

**Figure 2:**
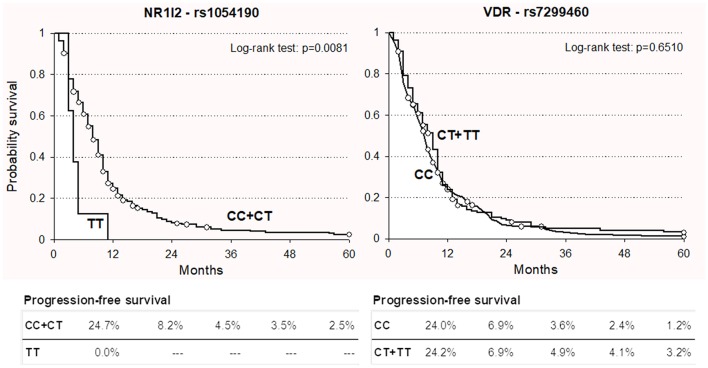
Kaplan-Meier estimates of progression-free survival according to selected *NR1I2*-rs1054190 and *VDR*-rs7299460 polymorphisms in combined discovery and replication cohorts (*n* = 337).

For both polymorphisms, the impact on PFS was consistent with the effect observed on OS. In particular, the *NR1I2* rs1054190-TT polymorphic genotype resulted in an increased risk of progression in both the discovery and replication cohorts, with a similar size effect (HR = 2.65 and HR = 2.67, respectively). These associations became significant in the discovery cohort (*P* = 0.0413). Kaplan-Meier curves for PFS according to *NR1I2*-rs1054190 in the combined discovery and replication cohorts are shown in Figure 2; patients carrying the minor allele homozygous rs1054190-TT genotype had a shorter PFS compared to CC or CT carriers (*P* = 0.0081 log-rank test). The *VDR*-rs7299460-T allele was predictive of a prolonged PFS in the discovery and replication set (HR = 0.93 and HR = 0.64, respectively). The association was not significant (*P* > 0.05; Figure 2 log-rank test *P* = 0.6510).

### Exploratory Analysis on a Set of Patients Treated With FOLFIRI Plus Bevacizumab

All the 74 samples were genotyped successfully. The main demographic and clinical characteristics of this study population (FOLFIRI plus bevacizumab group) are reported in Supplementary Table S3 and resulted well-matched with the discovery and replication cohorts (FOLFIRI group). At the end of the study follow-up, 49 patients (66%) were dead and 62 patients (84%) had tumor progression. Even if not significant, the impact of *NR1I2*-rs1054190 on OS and PFS was consistent with the effect observed in the discovery and replication sets. Particularly the *NR1I2* rs1054190-TT polymorphic genotype was associated with a poor prognosis in term of OS (HR = 2.82, 95% CI: 0.54–14.70, *P* = 0.2192) and PFS (HR = 1.39, 95% CI: 0.28–6.77, *P* = 0.6872). No significant association was observed for the *VDR*-rs7299460 variant.

## Discussion

Chemotherapy regimens, including FOLFIRI, are still the backbone of therapeutic strategies for mCRC. The combination of chemotherapeutic regimens with targeted therapy based on anti-angiogenic or anti-EGFR agents has further improved the survival of patients with mCRC, and great efforts have been made to identify potential predictive markers, such as molecular alterations or clinical characteristics (25–27).

A personalized medicine approach aimed at identifying genetic markers that predict which patients will benefit more from FOLFIRI therapy has the potential to improve treatment efficacy and survival, increasing patients' quality of life and reducing medical costs. In the last few years, great pharmacogenetic research efforts have been made to discover reliable predictors of the clinical outcome of mCRC patients treated with FOLFIRI-based therapy administration (8). However, while some polymorphisms (i.e., UGT1A1^*^28, *DPYD* variants) have been validated as predictors of irinotecan- or fluoropyrimidine-related toxicity and have been translated into specific clinical guidelines (10), no validated germline markers that could predict the response to therapy and patients survival have been still identified (8, 11).

The main finding of the present study was the identification of *NR1I2*-rs1054190 polymorphism as prognostic markers of OS, with a consistent effect in two independent cohorts of patients homogeneously treated with FOLFIRI regimen. Patients carrying the homozygous polymorphic *NR1I2* rs1054190-TT genotype exhibited a significantly shorter median OS and increased risk of disease progression to FOLFIRI treatment compared to the other patients.

*NR1I2* encodes for PXR, the most studied member of the NR family in an oncology setting, which could be defined as a xenosensor, due to its activity as a mediator between environmental stimuli and gene expression, with specific regard to drug-transforming genes (13–15). PXR is highly expressed in pharmacologically relevant organs, such as the liver, intestinal tract, and kidney, as well as in many solid cancers, including CRC, with a local effect on the expression of several phase I/II enzymes, ATP-binding cassette (ABC), and solute carrier (SLC) membrane transporters (13–15, 28). PXR over-expression in colon cancer cell lines and tumor biopsies was previously associated with irinotecan resistance due to increased inactivation of the active metabolite SN-38 through UGT1A induction (29). SN-38 has also been indicated to induce PXR, leading to increased irinotecan metabolism mediated by UGT1As, cytochromes (i.e., CYP3A4, CYP3A5), and ABC transporters (30). Recently, some *PXR* polymorphisms (i.e., rs10934498 and rs2472677) were shown to significantly affect exposure to SN-38 and therapeutic outcomes in advanced CRC patients receiving irinotecan (31). *In vitro* data further suggest that PXR is involved, through the modulation of an efflux carrier [e.g., multidrug resistance-associated protein 3 (MRP3)], in the determination of pharmacoresistance toward 5-FU, which is used with irinotecan in the FOLFIRI regimen (32).

The rs1054190 variant is located in the 3' untranslated region (UTR) of *NR1I2* and was previously suggested to impact on the PXR expression level by literature data. Particularly, an *ex vivo* analysis on head and neck squamous cell carcinoma tissue samples, highlighted that the presence of the rs1054190 variant T allele was associated with a reduced protein expression level, quantified by immunohistochemistry (33). Another study tried to elucidate how the rs1054190 variant leads to an alteration of the PXR expression. This polymorphism was predicted by an *in silico* analysis to alter a miRNA binding site, affecting microRNA-mediated PXR regulation. In particular, the C to T base change was predicted to modify the binding affinity of existing binding sites for a panel of microRNAs. This variation in the microRNA-mRNA binding efficiency could disrupt normal post-transcriptional PXR regulation, altering the level of PXR expression in a tissue-specific manner (34). A functional prediction was also performed in the present study using some online tools (i.e., HaploReg v4.1; RegulomeDB v1.1). The bioinformatic analysis, summarized in Supplementary Table S4A, further confirmed the potential impact of the rs1054190 T variant on the PXR expression [three quantitative trait loci (QTL) hit by Haploreg; RegulomeDB score of 5 that means ‘minimal binding evidence' supported by transcription factors binding or DNase peak data]. Thus, altered PXR expression due to deregulated post-transcriptional control may impact the trans-activation of downstream FOLFIRI-related ADME proteins. The resulting changes in the pharmacological profile could reduce therapeutic effectiveness and, thus, patient survival. On the other hand, we cannot exclude that the observed impact of the *NR1I2* rs1054190-TT genotype on OS could also be due to intrinsic differential tumor aggressiveness related to the patient genetic background. Emerging *in vivo* and *in vitro* data suggest that PXR is a key regulator of tumor cell proliferation and apoptosis, promoting a malignant phenotype (35). In particular, the activation of PXR in human colon tumor cell lines and xenograft models has been shown to enhance cell growth, invasion, and metastasis through different molecular mechanisms (i.e., PXR-mediated FGF19 and p53 signaling). Nuclear PXR expression also correlates with the clinical state of primary human colon cancer, significantly impacting patient survival (36–38). Taking these data into consideration, the significant effect of the *NR1I2* rs1054190-TT genotype on patient survival in the present study was probably the consequence of broad and multifactorial involvement of PXR in CRC biology and pharmacology. Further investigations will be required to better understand the exact biological mechanisms underlying the observed clinical associations.

Another novel result emerged from the present work is the prognostic role of the *VDR*-rs7299460 polymorphism that was significantly related to OS. Beyond its physiological role in calcium and phosphate homeostasis, VDR, another member of the NR family, has been demonstrated to cooperate in the transcriptional regulation of ADME genes (i.e., *CYPs, UGT1As, ABC/SLC transporters*), possibly affecting the drug disposition profile and coordinating key cellular processes, such as cell differentiation, modulation of inflammation, apoptosis, cell proliferation, invasion and metastatic processes, and angiogenesis (39, 40). In the current work, patients harboring at least one polymorphic rs7299460-T allele presented with longer median OS than those with the wild-type CC genotype. The phenotypic consequence of this intronic polymorphism is still unknown and no literature data are available until now. In the present study, an *in silico* functional prediction analysis was performed using some online tools (i.e., HaploReg v4.1; RegulomeDB v1.1). This analysis, summarized in Supplementary Table S4B, suggested that the rs7299460 variant could potentially affect the chromatin architecture, nucleosomal positioning, DNA methylation pattern and ultimately the accessibility to DNA for gene transcription; moreover this polymorphism resulted DNAse hypersensitive and is located in transcriptional binding element (3 altered motifs) with a consequential impact on the regulation of *VDR* expression (2QTL hits by HaploReg). The predicted functional effect was summed up by the RegulomeDB score that is equal to 5, meaning “minimal binding evidence” supported by transcription factors binding or DNase peak data. Rs7299460 is also a tagging polymorphism of other two intronic variations located in the same haploblock (*r*^2^ ≥ 0.8) (i.e., rs7136534, *r*^2^ = 0.82; rs10083198, *r*^2^ = 1), with a predicted impact on protein functionality. It should not be excluded that these additional linked variants could be responsible for the observed clinical phenotype.

The reported prognostic effect of *VDR*-rs7299460 is consistent with literature reports. Previous prospective epidemiological studies performed in various solid cancers (i.e., pancreatic cancer, melanoma, and prostate cancer) demonstrated a strong association between the minor allele *VDR* rs7299460-T and longer OS or cancer-specific survival (41–43). In addition to the contribution to ADME gene regulation, VDR also exerts an effect on cancer biology and the cell proliferation capacity, which could explain its impact on patient survival (41–43). The onco-protective action of VDR and its ligand (vitamin D) has largely been reported in CRC (40, 44, 45). VDR can improve colorectal cancer prognosis by inducing differentiation, promoting apoptosis, inhibiting cancer development and growth, and decreasing cancer cell proliferation and tumor angiogenesis (40, 44, 45). In summary, considering the reported prognostic effect of VDR-rs7299460 and its putative effect on gene transcription, this variant could be speculated to lead to higher VDR expression and improved patient survival by probably affecting the tumor biology and aggressiveness. However, further functional analysis will be required to test this hypothesis.

Some limitations of the present study need to be considered. First, although FOLFIRI remains the backbone of mCRC therapy, the current standard treatment for mCRC patients is no longer chemotherapy alone. The combination of chemotherapeutic regimens with anti-angiogenic or anti-EGFR agents has further improved the survival of mCRC patients and research efforts have been made to identify potential predictive markers both from molecular and clinical point of view. Particularly, some somatic alterations, such as *KRAS, NRAS*, and *BRAF* mutations, showed to play a role in predicting the response to EGFR-targeted therapy and/or patients prognosis (25, 26, 46). Moreover, the microsatellite instability /mismatch repair (MSI/MMR) status of the mCRC tumors have been recently shown to impact the response to the immunotherapy agents (47). Among the clinical characteristics, the primary tumor site demonstrated to have both a prognostic value and a predictive power in patients with RAS wild-type mCRC (48). However, despite the many efforts that have been made so far in identifying molecular and clinical factors that could discriminate mCRC patients with different response to therapy and prognosis, a significant variability in the clinical outcome is still present. Therefore the evaluation of the host genetic profile could contribute to better stratify patients who undergo therapy for mCRC on the basis of the treatment outcome. Our findings suggest better chemo-responsiveness in some patients depending on their inhered genetic features, and this is expected to be independent from the association with a targeted drug. This hypothesis was partly supported by our exploratory analysis on a small cohort of patients treated with FOLFIRI in combination with bevacizumab that showed for the *NR1I2*-rs1054190 marker a concordant effect on the clinical outcome with that observed in the FOLFIRI-treated group. In this patients cohort, the size effect resulted more limited probably due to the lower sample size. This promising but only preliminary observation calls for additional studies aiming to define the effect of NRs markers on FOLFIRI outcome in a clinical context in which FOLFIRI is used in association with anti-angiogenic or anti-EGFR agents. Second, the genetic analysis was performed retrospectively on prospective cohorts, which implies the need to perform further independent biomarker-driven prospective clinical trials to validate our results. Moreover, the clinical impact of the genetic markers emerged in the present work based on FOLFIRI treated patients should be evaluate in other chemotherapeutic regimens administered in the first-line setting as FOLFOX (oxaliplatin, fluorouracil, leucovorin) or FOLFOXIRI (oxaliplatin, irinotecan, fluorouracil, leucovorin) used alone or in combination with targeted agents. Third, the lack of data on the MSH status and somatic alterations (i.e., the *KRAS, NRAS*, and *BRAF* status) of the tumors included in the analysis could have affected the interpretation of the results. Furthermore, the exact functional meanings of the markers identified in the present study are still unknown, and formal functional analyses should be performed to better understand the molecular mechanism underlying the observed associations. However, the clinical association reported in the current paper, together with *in silico* data and evidence in the literature, supports a potential phenotypic impact of these variants on the level of NR expression. Another limitation of the present study is that it focused only on common genetic variants with MAF ≥ 0.05. As pointed out recently, rarer genetic variants could account for a high percentage of inter-individual variability in drug metabolism, including NR genes (49, 50), and for the observed inter-individual heterogeneity in drug pharmacokinetics. Therefore, future pharmacogenetic approaches should include these emerging markers to better describe patient phenotypes regarding the response to pharmacological treatment.

**Figure 3:**
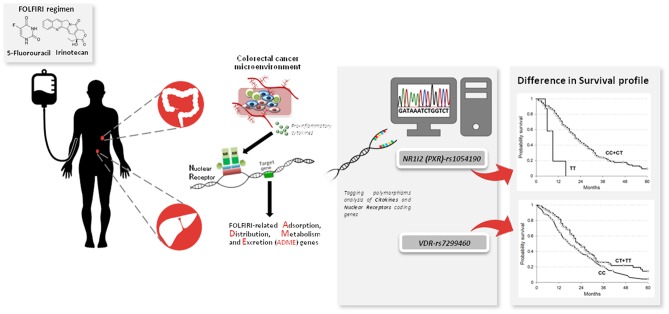
NR1I2 (PXR)-rs1054190 and VDR-rs7299460 polymorphisms as novel pharmacogenetic prognostic markers to personalize the treatment of metastatic colorectal cancer (mCRC) patients treated with a combination of irinotecan and fluoropyrimidines (FOLFIRI). Nuclear receptors (NRs) coding genes together with other inflammation-related genes have been described as mediators of cancer-related inflammation stimuli and gene expression profile, with specific regard to drugs transforming genes. Pharmacogenetic analysis was performed focusing on NRs and cytokine genes in CRC patients treated with FOLFIRI. NR1I2(PXR)-rs1054190 and VDR-rs7299460 polymorphisms arose as prognostic markers of patient survival and could be considered to optimize FOLFIRI-based treatment of mCRC patients.

The present study demonstrated for the first time that *NR1I2*-rs1054190 and *VDR*-rs7299460 polymorphisms are associated with the prognosis of mCRC patients in term of OS and PFS. Considering the increasing number of therapeutic options available for the treatment of mCRC, some rational criteria for selecting the best treatment for each patient are greatly needed. The present results suggest that genetic variants related to ADME gene regulators could be helpful for identifying patients who are most likely to benefit from chemotherapy. A visual summary of the main findings of the article could be find at Figure 3. Rational and precise selection of the most effective anti-cancer treatment has the potential not only to improve mCRC patient survival and quality of life, but also to reduce medical costs.

## Data Availability Statement

The raw data supporting the conclusions of this manuscript will be made available by the authors, without undue reservation, to any qualified researcher.

## Ethics Statement

The studies involving human participants were reviewed and approved by Comitato Etico Indipendente-Centro di Riferimento Oncologico di Aviano CHU de Quebec ethics committees. The patients/participants provided their written informed consent to participate in this study.

## Author Contributions

EDe and EC contributed to designing the study, writing the main manuscript, and elaborating the tables and figures. JP was involved in the statistical analysis and interpretation of data. AL participated in the molecular analysis for replication cohort. CG participated in the enrollment of the replication patient group and the collection of clinical and genotyping data on this set. RR, ABi, EDr, and LR participated in the molecular analysis for the discovery cohort. MG, ABu, and MD'A participated in the enrollment of the discovery patient group and the collection of clinical data. EL, DJ, and FC participated in the design, enrolment of the replication cohort, and the collection of clinical data. GT was the guarantor. All authors reviewed the manuscript.

### Conflict of Interest

The authors declare that the research was conducted in the absence of any commercial or financial relationships that could be construed as a potential conflict of interest.

## Abbreviations

ABC: ATP-binding cassette
ADME, adsorption, distribution, metabolism: and excretion
ADT: Assay Design Tool
CI: confidence interval
DPYD: dihydropyrimidine dehydrogenase
FOLFIRI, irinotecan: 5-fluorouracil and leucovorin
HNF1A: hepatocyte nuclear factor 1 alpha
HR: hazard ratio
LD: linkage disequilibrium
MAFs: minor allele frequencies
mCRC: metastatic colorectal cancer
NR: nuclear receptors
OS: overall survival
PFS: progression-free survival
QTL: quantitative trait loci
SLC: solute carrier
STAT-3: signal transducer and activator of transcription 3
TagSNP: tagging polymorphism
UGT1A1: UDP glucuronosyl transferase family 1 member A1
UTR: 3’
untranslated region
VDR: vitamin D receptor.
