# Supplementary material for: Germline Polymorphisms in the Nuclear Receptors PXR and VDR as Novel Prognostic Markers in Metastatic Colorectal Cancer Patients Treated With FOLFIRI
Source: Front Oncol. 2019 Nov 26;9:1312. doi: 10.3389/fonc.2019.01312 (PMC6901926; doi:10.3389/fonc.2019.01312)
Supplement: Supplementary file 1 [file Table_1.docx]

Title: Germline polymorphisms in the nuclear receptors PXR and VDR as novel prognostic markers in metastatic colorectal cancer patients treated with FOLFIRI

**Authors:** Elena De Mattia^1^*, Jerry Polesel^2^, Rossana Roncato^1^, Adrien Labriet^3^, Alessia Bignucolo^1^, Eva Dreussi^1^, Loredana Romanato^1^, Michela Guardascione^1^, Angela Buonadonna^4^, Mario D’Andrea^5^, Eric Lévesque^6^, Derek Jonker^7^, Félix Couture^6^, Chantal Guillemette^3^, Erika Cecchin^1^#, Giuseppe Toffoli^1*^#

#Cecchin E. and Toffoli G. share last authorship

**Correspondence to:**

*Dr. Elena De Mattia PhD, Clinical and Experimental Pharmacology, CRO- National Cancer Institute, Via Franco Gallini n. 2, 33081 Aviano (PN) –Italy. [edemattia@cro.it](mailto:edemattia@cro.it)

**Supplementary Table S1. Distribution of mCRC patients from the discovery (n=247) and replication (n=90) cohorts according to gene polymorphisms (SNP).**

| **Genes** | **SNP** | **Base change** | **Discovery cohort** | | | **Replication cohort** | | |
| --- | --- | --- | --- | --- | --- | --- | --- | --- |
|  |  |  | **AA** | **Aa** | **aa** | **AA** | **Aa** | **aa** |
|  |  |  |  |  |  |  |  |  |
| *HNF4A* | rs3212208 | T>C | 0.8502 | 0.1457 | 0.0040 | 0.8111 | 0.1667 | 0.0222 |
| *NFKB1* | rs3774934 | G>A | 0.7202 | 0.2634 | 0.0165 | 0.8556 | 0.1414 | 0.0000 |
| *NR1I2* (PXR) | rs1054190 | C>T | 0.7409 | 0.2389 | 0.0202 | 0.7889 | 0.1778 | 0.0333 |
| *NR1I2* (PXR) | rs6784598 | C>G | 0.3862 | 0.4675 | 0.1463 | 0.3778 | 0.4333 | 0.1889 |
| *NR1I3* (CAR) | rs4073054 | T>G | 0.3918 | 0.4286 | 0.1796 | 0.4444 | 0.4444 | 0.1111 |
| *PPARA* | rs4253655 | G>A | 0.6382 | 0.3496 | 0.0122 | 0.5169 | 0.4494 | 0.0037 |
| *PPARD* | rs4713854 | A>C | 0.8502 | 0.1457 | 0.0040 | 0.7865 | 0.2022 | 0.0112 |
| *PPARG* | rs7626560§ | C>T | 0.7045 | 0.2713 | 0.0243 | 0.6889 | 0.2556 | 0.0556 |
| *STAT3* | rs17593222 | C>G | 0.8947 | 0.1012 | 0.0040 | 0.8090 | 0.1798 | 0.0112 |
| *VDR* | rs4760648 | C>T | 0.3347 | 0.4898 | 0.1755 | 0.3889 | 0.5000 | 0.1111 |
| *VDR* | rs7299460 | C>T | 0.4453 | 0.4291 | 0.1255 | 0.5333 | 0.4000 | 0.0667 |
|  |  |  |  |  |  |  |  |  |

§rs7626560 was replace in the replication set by rs13099078 which is in complete Linkage Disequilibrium (r2=1)
