# Supplementary material for: Germline Polymorphisms in the Nuclear Receptors PXR and VDR as Novel Prognostic Markers in Metastatic Colorectal Cancer Patients Treated With FOLFIRI
Source: Front Oncol. 2019 Nov 26;9:1312. doi: 10.3389/fonc.2019.01312 (PMC6901926; doi:10.3389/fonc.2019.01312)
Supplement: Supplementary file 2 [file Table_2.docx]

Title: Germline polymorphisms in the nuclear receptors PXR and VDR as novel prognostic markers in metastatic colorectal cancer patients treated with FOLFIRI

**Authors:** Elena De Mattia^1^*, Jerry Polesel^2^, Rossana Roncato^1^, Adrien Labriet^3^, Alessia Bignucolo^1^, Eva Dreussi^1^, Loredana Romanato^1^, Michela Guardascione^1^, Angela Buonadonna^4^, Mario D’Andrea^5^, Eric Lévesque^6^, Derek Jonker^7^, Félix Couture^6^, Chantal Guillemette^3^, Erika Cecchin^1^#, Giuseppe Toffoli^1*^#

#Cecchin E. and Toffoli G. share last authorship

**Correspondence to:**

*Dr. Elena De Mattia PhD, Clinical and Experimental Pharmacology, CRO- National Cancer Institute, Via Franco Gallini n. 2, 33081 Aviano (PN) –Italy. [edemattia@cro.it](mailto:edemattia@cro.it)

**Supplementary Table S2. Clinical-demographic features and survival profile of the patients carrying the *NR1I2* (PXR) rs1054190-TT genotype.**

| **Patients** | **Age** | **Gender** | **Cancer Site** | **Status Alive/Dead** | | **Survival (months)** |
| --- | --- | --- | --- | --- | --- | --- |
|  |  |  |  | **1year** | **2years** |  |
| 1-discovery | 46 | female | rectum | Dead | Dead | 10 |
| 2-discovery | 35 | female | rectum | Dead | Dead | 6 |
| 3-discovery | 57 | male | right colon | Dead | Dead | 7 |
| 4-discovery | 55 | female | right colon | Lost to follow-up | Lost to follow-up | 5 |
| 5-discovery | 65 | female | left colon | Lost to follow-up | Lost to follow-up | 8 |
| 6-replication | 43 | male | rectum | Dead | Dead | 9 |
| 7-replication | 75 | male | right colon | Alive | Dead | 16 |
| 8-replication | 36 | male | left colon | Dead | Dead | 6 |
