# Supplementary material for: Germline Polymorphisms in the Nuclear Receptors PXR and VDR as Novel Prognostic Markers in Metastatic Colorectal Cancer Patients Treated With FOLFIRI
Source: Front Oncol. 2019 Nov 26;9:1312. doi: 10.3389/fonc.2019.01312 (PMC6901926; doi:10.3389/fonc.2019.01312)
Supplement: Supplementary file 3 [file Table_3.docx]

Title: Germline polymorphisms in the nuclear receptors PXR and VDR as novel prognostic markers in metastatic colorectal cancer patients treated with FOLFIRI

**Authors:** Elena De Mattia^1^*, Jerry Polesel^2^, Rossana Roncato^1^, Adrien Labriet^3^, Alessia Bignucolo^1^, Eva Dreussi^1^, Loredana Romanato^1^, Michela Guardascione^1^, Angela Buonadonna^4^, Mario D’Andrea^5^, Eric Lévesque^6^, Derek Jonker^7^, Félix Couture^6^, Chantal Guillemette^3^, Erika Cecchin^1^#, Giuseppe Toffoli^1*^#

#Cecchin E. and Toffoli G. share last authorship

**Correspondence to:**

*Dr. Elena De Mattia PhD, Clinical and Experimental Pharmacology, CRO- National Cancer Institute, Via Franco Gallini n. 2, 33081 Aviano (PN) –Italy. [edemattia@cro.it](mailto:edemattia@cro.it)

**Supplementary Table 3. Socio-demographic and clinical characteristics of patients enrolled in the exploratory cohort.**

|  | **FOLFIRI + Bevacizumab (n=74)** | |
| --- | --- | --- |
|  | **N** | **(%)** |
|  |  |  |
| Gender |  |  |
| Male | 48 | (64.9) |
| Female | 26 | (35.1) |
| Age (years) |  |  |
| <55 | 19 | (25.7) |
| 55-59 | 9 | (12.2) |
| 60-64 | 14 | (18.9) |
| ≥65 | 32 | (43.2) |
| Cancer site |  |  |
| Right colon | 20 | (27.0) |
| Left colon/Rectum | 47 | (63.5) |
| Colon, NOS | 7 | (9.5) |
| Stage at cancer diagnosis |  |  |
| I-II | - |  |
| III | - |  |
| IV | - |  |
| Radical surgery |  |  |
| No | - |  |
| Yes | - |  |
| Adjuvant therapy |  |  |
| No | - |  |
| Yes | - |  |
| Median follow-up (Q1-Q3) | 27 (15-36) | |
| Overall survival (95% CI) |  |  |
| 1 year | 84.9% (74.4%-91.4%) | |
| 2 years | 56.2% (44.1%-66.6%) | |
| 3 years | 35.6% (24.9%-46.5%) | |
|  |  |  |

## Abbreviation: NOS, Not Otherwise Specified
