# Supplementary material for: Germline Polymorphisms in the Nuclear Receptors PXR and VDR as Novel Prognostic Markers in Metastatic Colorectal Cancer Patients Treated With FOLFIRI
Source: Front Oncol. 2019 Nov 26;9:1312. doi: 10.3389/fonc.2019.01312 (PMC6901926; doi:10.3389/fonc.2019.01312)
Supplement: Supplementary file 4 [file Table_4.docx]

Title: Germline polymorphisms in the nuclear receptors PXR and VDR as novel prognostic markers in metastatic colorectal cancer patients treated with FOLFIRI

**Authors:** Elena De Mattia^1^*, Jerry Polesel^2^, Rossana Roncato^1^, Adrien Labriet^3^, Alessia Bignucolo^1^, Eva Dreussi^1^, Loredana Romanato^1^, Michela Guardascione^1^, Angela Buonadonna^4^, Mario D’Andrea^5^, Eric Lévesque^6^, Derek Jonker^7^, Félix Couture^6^, Chantal Guillemette^3^, Erika Cecchin^1^#, Giuseppe Toffoli^1*^#

#Cecchin E. and Toffoli G. share last authorship

**Correspondence to:**

*Dr. Elena De Mattia PhD, Clinical and Experimental Pharmacology, CRO- National Cancer Institute, Via Franco Gallini n. 2, 33081 Aviano (PN) –Italy. [edemattia@cro.it](mailto:edemattia@cro.it)

**Supplementary Table S4**: *In silico* predicted functional effect of polymorphisms in the **A)** *NR1I2*-rs1054190 and **B)** *VDR*-rs7299460 haploblocks by HaploReg v.4.1 and RegulomeDB v1.1. Targeted marker is evidenced in red.

| **General data** | | | | **HaploReg*** | | | | | | | **RegulomeDB Score**** |
| --- | --- | --- | --- | --- | --- | --- | --- | --- | --- | --- | --- |
| **dbSNP ID** | **Chromosome Location (GRCh38)** | **LD (r^2^)** | **SNP Location** | **Promoter histone marks** | **Enhancer histone marks** | **DNAse** | **Proteins bound** | **Motifs changed** | **GRASP QTL hits** | **Selected**  **eQTL hits** |  |
| **A) *NR1I2*-rs1054190 haploblock** | | | | | | | | | | | |
| [rs11929668](https://pubs.broadinstitute.org/mammals/haploreg/detail_v4.1.php?query=&id=rs11929668) | chr3:119816948 | 0.8 | NR1I2 (intronic) |  | BRST, SKIN | SKIN,MUS |  | GATA,Pax-5,p53 |  | 2 hits | 5 |
| [rs10511395](https://pubs.broadinstitute.org/mammals/haploreg/detail_v4.1.php?query=&id=rs10511395) | chr3:119817712 | 0.8 | NR1I2 (3'UTR) |  |  |  |  | AP-2,Nr2f2,Zfx |  | 2 hits | 5 |
| [**rs1054190**](https://pubs.broadinstitute.org/mammals/haploreg/detail_v4.1.php?query=&id=rs1054190) | **chr3:119817871** | **1** | **NR1I2 (3'UTR)** |  |  |  |  |  | **1 hit** | **2 hits** | **5** |
| [rs1054191](https://pubs.broadinstitute.org/mammals/haploreg/detail_v4.1.php?query=&id=rs1054191) | chr3:119818050 | 0.8 | NR1I2 (3'UTR) |  |  |  |  | Rad21 |  | 2 hits | 4 |
| **B) *VDR*-rs7299460 haploblock** | | | | | | | | | | | |
| [rs7136534](https://pubs.broadinstitute.org/mammals/haploreg/detail_v4.1.php?query=&id=rs7136534) | chr12:47900843 | 0.82 | VDR (intronic) | BRST, BLD, GI | 20 tissues | 19 tissues | 7 bound proteins | 4 altered motifs |  | 2 hits | 4 |
| [rs10083198](https://pubs.broadinstitute.org/mammals/haploreg/detail_v4.1.php?query=&id=rs10083198) | chr12:47902182 | 1 | VDR (intronic) |  |  | GI,BLD |  | SRF |  | 1 hit | 5 |
| [**rs7299460**](https://pubs.broadinstitute.org/mammals/haploreg/detail_v4.1.php?query=&id=rs7299460) | **chr12:47902485** | **1** | **VDR (intronic)** | **6 tissues** | **12 tissues** | **GI** |  | **GATA,Pax-5,TAL1** |  | **2 hits** | **5** |

*ENCODE-HaploReg v4.1 (<https://pubs.broadinstitute.org/mammals/haploreg/haploreg.php>) was employed to test the functional effect of a selected polymorphism and all the others included in the same haploblock at a stringency of r^2^=0.80 using the linkage disequilibrium (LD) data from 1000 Genomes Project (EUR). HaploReg includes chromatin state and protein binding annotation from the Roadmap Epigenomics and the Encyclopedia of DNA Elements (ENCODE) projects, sequence conservation across mammals, the effect of polymorphisms on regulatory motifs, and the effect of polymorphisms on expression from expression quantitative trait locus (eQTL) studies.

**RegulomeDB v1.1 (<http://www.regulomedb.org/>) is a database that annotates polymorphisms in the intergenic regions of the human genome by integrating a big collection of regulatory information from several public dataset. This tool presents a score system with categories ranging from 1 to 6 with the lower score indicating the stronger evidence for a variant to be in a functional region; the score is assigned by integration annotation data on methylation profile, chromatin structure, protein motifs, binding to transcription factors and enhancer activity. Score: 4= TF binding + DNase peak (minimal binding evidence); 5=TF binding or DNase peak (minimal binding evidence).

Abbreviation: SNP, single nucleotide polymorphism; UTR, untranslated region.
